# Sunlight Intensity Based Global Positioning System for Near-Surface Underwater Sensors

**DOI:** 10.3390/s120201930

**Published:** 2012-02-10

**Authors:** Javier V. Gómez, Frode E. Sandnes, Borja Fernández

**Affiliations:** 1 Department of Systems and Automation, Carlos III University of Madrid, Avda. de la Universidad 30, 28911 Leganés, Madrid, Spain; E-Mail: jvgomez@pa.uc3m.es; 2 Institute of Information Technology, Faculty of Technology, Art and Design, Oslo and Akershus University College of Applied Sciences, P.O. Box 4, St. Olavs plass, N-0130 Oslo, Norway; 3 Department of Computer Science, Rey Juan Carlos University of Madrid, Calle Tulipán s/n, 28933 Móstoles, Madrid, Spain; E-Mail: b.fernandezo@alumnos.urjc.es

**Keywords:** outdoor, localization, light, intensity, global positioning, sunlight, underwater

## Abstract

Water monitoring is important in domains including documenting climate change, weather prediction and fishing. This paper presents a simple and energy efficient localization strategy for near surface buoy based sensors. Sensors can be dropped randomly in the ocean and thus self-calibrate in terms of geographic location such that geo-tagged observations of water quality can be made without the need for costly and energy consuming GPS-hardware. The strategy is based on nodes with an accurate clock and light sensors that can regularly sample the level of light intensity. The measurements are fitted into a celestial model of the earth motion around the sun. By identifying the trajectory of the sun across the skies one can accurately determine sunrise and sunset times, and thus extract the longitude and latitude of the sensor. Unlike previous localization techniques for underwater sensors, the current approach does not rely on stationary or mobile reference points.

## 1. Introduction

Most of the Earth’s surface is covered in water which affects us in numerous ways. We depend on the oceans as a vital source for food. Moreover, the conditions in the oceans greatly affect the weather on land. Increasingly, we have become aware that our actions also affect the world’s oceans and there is currently much focus on climate change. For instance, variations in ocean salinity are important as it is a vital indicator of water cycle and used for climate forecasting [1]. Several ocean-monitoring systems have been developed for collecting data about the upper ocean salinity and temperature data using ships and buoys. Not only are the measurements themselves important, but also the geographic location where the measurement is made. In addition, underwater or near surface sensors and sensor networks have been used for several other civilian and military applications such as salmon detection and tracking [2].

There is a vast body of research on underwater sensors and sensor networks. Issues addressed include energy consumption [3], image analysis [4–6], optics [7], routing protocols [8–10], sound [11] and acoustic localization through measurements [12]. In fact, localization is a challenging problem for several reasons. The commonly used GPS technology is dependent on energy consuming hardware, long satellite locking times and a satellite infrastructure that is approaching the end of its lifetime [13]. Moreover, another problem with GPS technology is that GPS signals do not propagate through water [14], and this is the reason existing research on underwater sensor networks focuses on acoustic communication. However, underwater conditions are often characterized by harsh physical conditions resulting in low bandwidth, large propagation delays and high bit error rates. Another challenge is the speed of sound due to water currents. Existing localization techniques thus try to combat these challenges by employing static or mobile reference nodes [15,16]. Hence, the localization mechanisms are dependent on a complex infrastructure of sensor nodes. This work proposes a localization technique that is not dependent on such infrastructure, but rather uses the sun as a reference point.

This paper is organized as follows. In Section 2 the proposed localization system is described, outlining hardware requirements and software algorithms. Next, Section 3 shows the simulation results and how they validate the model. Following, Section 4 contains the results of the system working with real sunlight data. In Section 5 the advantages and disadvantages are discussed. Last, Section 6 summarizes the conclusions obtained.

## 2. SGP System Description

The Sunlight Intensity based Global Positioning System (SGPS) is able to localize outdoor objects by its earth coordinates (longitude and latitude) using only light intensity information.

The system described takes into account stationary objects. This means that the object has to be in the same position during the whole day (or at least during the daylight time). Due to the accuracy of the system and due to the fact that the sunlight intensity can be considered constant in small areas, small movements are possible. In this case the term *small* depends on the accuracy of the system, a few kilometers.

### 2.1. System Requirements

The method proposed herein is based on electronic devices with a built-in clock which maintains a relatively accurate account of time and date. Digital clocks are built into most electronic hardware and can run for many years on one single battery with limited drift. It is therefore assumed that at any time the object can enquire the current time *t* and date *d*. It is assumed that the time is set to Universal Time (UTC) which makes the calculations presented herein simple and is represented in decimal form in the range from 0 to 24. Issues such as daylight saving time are thus avoided. Next, the date *d* is represented as the day of the year, where 1 January is day 1, *etc*.

Next, the strategy requires that the device has some form of light sensor that is capable of measuring the lighting condition *e*. The simplest possible device is a light intensity sensor which gives a voltage directly proportional to the incident light intensity. This type of sensors consume no electrical energy since they actually change the light energy into electrical energy without any power source. Some other devices could be used, for instance a simple and low cost exposure value meter such as those built into most low-cost digital cameras [17–19] or it could be a low cost camera (CCD-chip). In the cases of exposure values (EV) then *e* is a real value between 0 to approximately 20 [20,21]. If a camera is used a simple representation of exposure can be obtained simply using:

(1) $$e=\frac{1}{\mathrm{XY}}\sum_{x=1}^{X}\sum_{y=1}^{Y}I_{x,y}$$

where *I_x,y_* is the pixel intensity for the pixel located at (*x, y*) in the image and *X* and *Y* are the numbers of image pixels in columns and rows correspondingly. To reduce computation a small subset of these points can be sampled throughout the image using some two dimensional sampling pattern.

The system proposed in this paper is designed to be as simple as possible. Therefore, apart from the light sensor, a microprocessor is required in order to analyze the data given by the sensor. This element consumes most of the energy in the system.

A feasible option for implementing the proposed system is to use *mote* technology [22]. These motes are small, low-cost electronic devices which includes sensor support, small microprocessors, wireless and serial communications. This kind of technology is being used during the last years in sensor networks. Furthermore, some of these motes are designed to be energy-efficient and their energy consumption allows to use these devices with single batteries for a long time.

**Figure 1.**
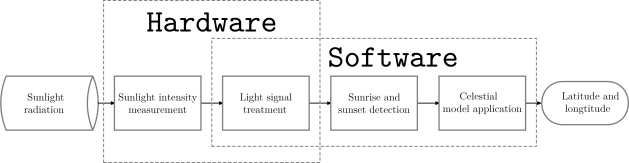
SGPS flowchart showing what steps are done by hardware and what by software.

**Figure 2.**
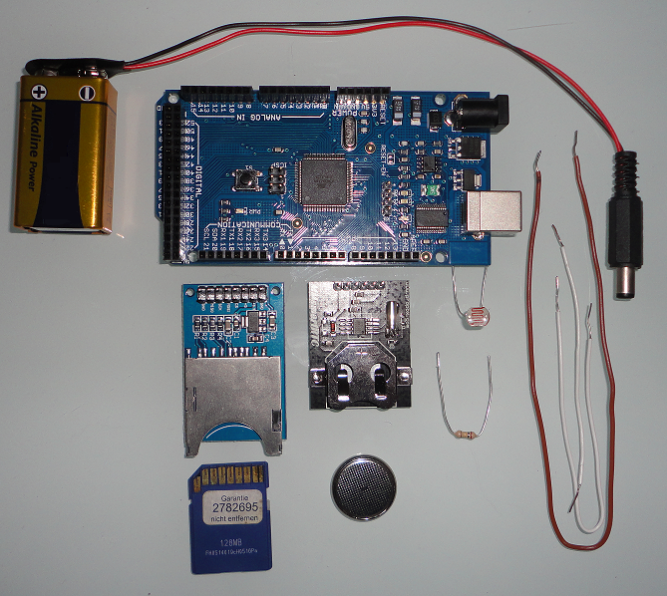
Hardware implementation based on Arduino Mega: power source, SD storage, clock and light dependent resistor (LDR) sensor. The simplest SGPS implementation costs less than US $40 in components.

The SGPS operation is summarized in the flowchart presented in Figure 1. Also, an Arduino-based implementation is suggested in Figure 2. The system can be designed in a more complex way, including other data measuring devices or using a more powerful microprocessor, but the system proposed herein is focused in a simple measuring strategy, with low-cost, energy-efficient components.

### 2.2. Celestial Model

**Figure 3.**
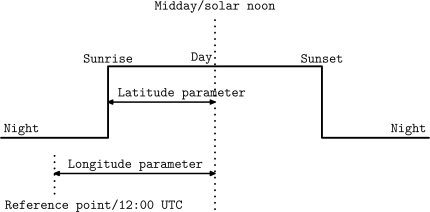
Daylight parameters influenced by latitude and longitude.

This paper presents a new algorithm to localize outdoor objects using only sunlight intensity data for a given location. With this information, it is possible to obtain the sunrise and sunset times for that location. Only with these two values, the celestial model determines the coordinates (both longitude and latitude). Thus, the coordinates can be expressed as a function of the sunrise and sunset times as shown in Figure 3.

There is an accurate, well-known algorithm based on Julian Day which allows to find out the sunrise and sunset times for a specific place by only knowing the coordinates of that place and also the date [23]. However, it is not possible to obtain the *inverse* of the algorithm since some formulas of this algorithm depend on both latitude and longitude, and when they are tried to be separated the result is that the latitude depends on the longitude and *vice-versa*.

Then, the method described herein can be reduced to the problem of identifying the sunset and sunrise times for a given day. Given an accurate measurement of the sunrise time *t_sunrise_* and sunset time *t_sunset_*, the solar noon *t_midday_* is simply:

(2) $$t_{\mathrm{midday}}=\frac{t_{\mathrm{sunrise}}+t_{\mathrm{sunset}}}{2}$$

The UTC representation of time assumes that sunrise occurs before sunset. If the sunset occurs before the sunrise within the 24 h UTC time window then it means that it is a fractional day, since the sunrise happens the day before (in UTC format) that sunset. This has to be taken into account when applying this equation, since if the equation is applied directly with a sunset time earlier than the sunrise time, the noon time will not be correct (in fact, the result would be the *midnight* time). Section 3.1 details how to overcome this issue when applying Equation (2).

As stated in Section 2.1 the times have to be expressed in UTC and in decimal format (from 0 to 24). For the next formulas all the times will be expressed in this format and the angles in radians. The angular sunset can be computed as follows:

(3) $$a_{\mathrm{sunset}}=\frac{\pi }{12}|t_{\mathrm{sunset}}-t_{\mathrm{midday}}|$$

and the declination of the sun *δ* can be approximated by the following Fourier series [24]:

(4) $$\begin{array}{c} \delta =0.006918-0.399912\;\text{cos}(\beta )+0.070257\;\text{sin}(\beta )\\ -0.006758\;\text{cos}(2\beta )+0.000907\;\text{sin}(2\beta )\\ -0.002697\;\text{cos}(3\beta )+0.00148\;\text{sin}(3\beta ) \end{array}$$

where *β* is the fractional year expressed in radians given by:

(5) $$\beta =\frac{2\pi }{365}\;d$$

and *d* is the day from 1 to 365. In the case of a leap-year, where *d* can be 366 the value for *β* is close to 2*π*, which gives the same result in the equation for *δ* as when *d* is 1 and *β* is equal to 2*π*/365.

Finally, the coordinates can be obtained. For the longitude *λ* (in radians), where positive values represent east and negative values represent west, the next equation is employed:

(6) $$\lambda =2\pi \;\frac{12-t_{\mathrm{midday}}}{24}$$

and the latitude *φ* (in radians) of the objects location can be found by numerically solving for latitude using the following equation, where positive values represent north and negative values represent south:

(7) $$\text{cos}(a_{\mathrm{sunset}})=\frac{\text{sin}(-0.0145)-\text{sin}(\delta )\;\text{sin}(\varphi )}{\text{cos}(\delta )\;\text{cos}(\varphi )}$$

The equations provided in this sections allows to find out the coordinates for outdoor objects by means of the times of the sunrise and the sunset for a given day.

### 2.3. Measurement Strategy

The system is designed to work autonomously, being able to localize itself within the first 24 h of being switched on. This implies that the system can determine its coordinates without any previous information besides the time.

Given a sufficient supply of electric power, for instance if the object has a steady power supply, the initialization can be simply performed using brute force by continuously sampling the lighting condition. If the light sensor is sampled at *r* samples per second, then this is the same as *M* samples for each 24 h cycle, given by:

M=24×60×60×r

The accuracy of the measurements will therefore be in the range of:

$$a=\frac{360}{M}$$

It will take maximum 24 h to identify the location and the effort involved is defined by *E* = *M* × *p*, where *p* is the energy consumed to perform each sample and *E* is the total energy. This is the optimal solution in terms of speed and accuracy. However, for a power constrained device the strategy is unrealistic as a very simple device could run out of power after just a few hours or sooner, although the latest technologies released are improving the energy consumption and some devices can run for months with standard off-the-shelf AA batteries.

### 2.4. Enhancements

**Figure 4.**
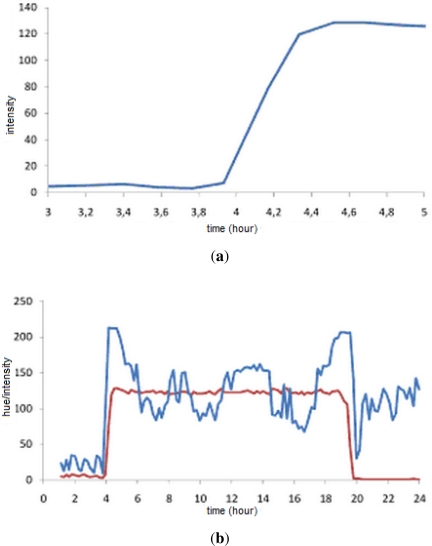
(**a**) Gradual increase in intensity towards sunset; (**b**) Sudden hue changes predict sunrises and sunsets.

Several theoretical enhancements are possible. Sunrises can be predicted if early measurements are taken. This is because the light intensity increases gradually over some time interval before one passes the threshold. If a light intensity measurement is taken that is above the night baseline value but yet below the threshold then this is a sign that a sunrise is approaching soon and the sample rate can be dynamically increased. This is illustrated by Figure 4(a) which shows an authentic intensity plot obtained using a webcam. Clearly the intensity rises for about 20 min before the sun breaks.

However, it may be more difficult to get a pre-warning of a sunset in this way as this will suddenly drop below the threshold value. One way to overcome this is to observe additional features. If the light sensor is capable of capturing color spectrum information then additional information can be exploited to better predict sunrises and sunsets. For instance, the CCD sensors in digital cameras are capable of detecting color as well as intensity. This is because sunsets and sunrises often are characterized by large changes in hue which affect entire scenes. Such changes in hue occur prior to sunsets and thus a detection in hue change can be used to predict an upcoming sunset. This is illustrated in Figure 4(a) which presents a 24 h plot obtained using a webcam. The hue *h*(*r, g, b*) of each pixel is computed from the *r*, *g* and *b* (red, green and blue) components as follows:

(8) $$h(r,g,b)=\mathrm{atan}2\;\left(2r-g-b,\;\sqrt{3}(g-b)\right)$$

The steady line shows the overall image intensity and the other line illustrates overall image hue. Clearly, the hue is changing dramatically just before the sunrise and sunset. In fact, these can be seen as the two peaks in the hue plot. However, the exploitation of such features is a topic of future research.

## 3. Simulation Results

In order to be able to evaluate the system independently of the problem of finding the sunrise and sunset time accurately, the algorithm proposed in Section 2.2 is tested with a theoretical celestial model. Thanks to this model, the sunrise and sunset times can be used as inputs to the system with a low error. Stationary objects are assumed since the objective of this section is to validate the celestial model and extract some conclusions prior to applying it to a real measurements.

The theoretical celestial model used in the simulations is based on [23], and to validate that these simulations are robust, they were benchmarked against a spreadsheet developed by the National Oceanic and Atmospheric Administration of the United States Department of Commerce (NOAA) [25].

**Figure 5.**
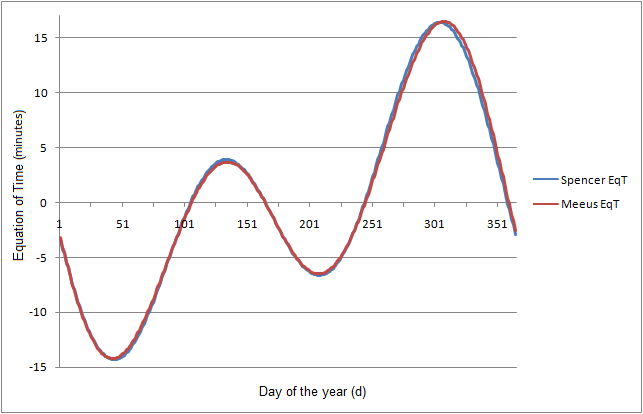
Comparison between the Meeus and Spencer Equations of Time.

Note that our system operates according to civil time (without taking into account the established time zones) while the NOAA calculations are based on solar times. The following equation can be used to convert between the two time-formats:

(9) $$t_{\mathrm{civil}}=t_{\mathrm{solar}}+\mathrm{EqT}$$

where the *EqT* value is the Equation of Time for a given day. There are different approaches for determining *EqT*. NOAA employs a very accurate expression given by Meeus [23]. However, this is a complex equation which uses parameters not available for this application. Thus, the expression proposed by Spencer [24] was employed since it only uses information about the day *d*:

(10) $$\begin{array}{ll} \mathrm{EqT}= & 229.18\times (0.000075+0.001868\;\text{cos}(\beta )\\ & -0.032077\;\text{sin}(\beta )-0.014615\;\text{cos}(2\beta )\\ & -0.040849\;\text{sin}(2\beta )) \end{array}$$

where *β* is described in Section 2.2. A comparison of the two equations of time is shown in Figure 5.

The NOAA sunrise and sunset time predictions have been verified to be accurate within a minute for locations between *±*72° latitude, and within 10 min outside those latitudes. Also, the celestial model employed is valid for dates between 1901 and 2099, due to approximations used in the Julian Day calculation.

The simulation involves finding the sunrise and sunset times, in UTC, using the NOAA approach for 2011. For each value the algorithm described in Section 2.2 is applied.

**Figure 6.**
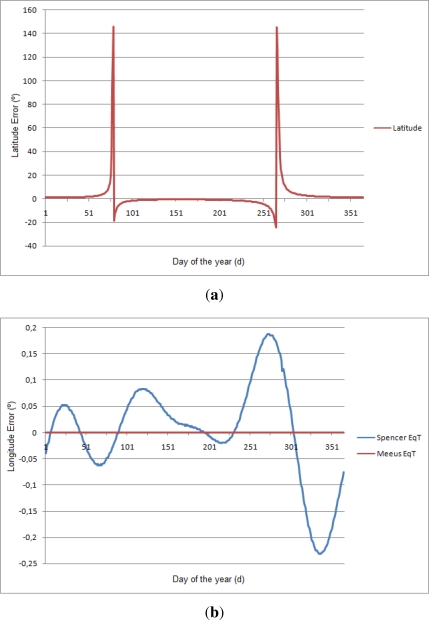
Simulation results of the SGPS using Oslo coordinates. (**a**) The latitude is independent of the Equation of Time; (**b**) The longitude error is zero when using the EqT value from the Meeus equation, but not when using the Spencer expression.

Figure 6 shows the results of this procedure using the Oslo (Norway) coordinates (59.95°N, 10.75°E). This figure shows that the system gives values closer to the coordinates used. The longitude is theoretically perfect with zero error. For the latitude, its results are accurate, but there are error peaks close to the equinoxes, where the value of the declination of the sun *δ* tends to 0. The equinoxes occur at 22, 23 March (*d* = 81, 82) and 20, 21 September (*d* = 265, 266).

One conclusion drawn from the simulation results is that the system works according to theory. The fact that the computational complexity is not critical allows to modify the algorithm, creating complex error compensation algorithms and even to use numerical solving in order to improve the accuracy.

### 3.1. Fractional Days

Section 2.2 mentioned that fractional days require special treatment for the algorithm to work. We define as fractional days those days where the sunset occurs before the sunset within the 24 h UTC time window.

For example, imagine that there is a day in which the sunrise occurs at 22 h and the sunset occurs at 4 h into the following day. Then, applying the algorithm midday is reported to occur at 13 h. This make no sense as midday occur at night. To avoid complex modifications of the algorithm the following strategy is employed: Midday of a fractional day within 24 h window is equal to the midday time calculated by the standard *t_midday_* Equation (1) using the sunrise of the previous day adding 12 h and obtaining the module of this number by 24. Or, more precisely:

(11) $$t_{\mathrm{md}}^{\prime }\left(d\right)=\mathrm{mod}\left(t_{\mathrm{md},\mathrm{frac}}\left(d\right)+12,\;24\right)$$

where

(12) $$t_{\mathrm{md},\mathrm{frac}}\left(d\right)=\frac{\mathrm{SR}(d-1)+\mathrm{SS}\left(d\right)}{2}$$

**Figure 7.**
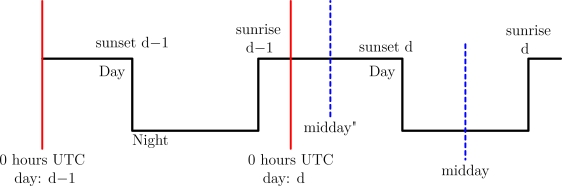
Correcting the midday error by the initial midday equation.

Applying this strategy to the previous example, in which the standard *t_midday_* equation gives a result of 13 h UTC, the new result yields a midday at 25 h UTC (13+12) or 1 h UTC of the same day (since 25 h UTC refers to *d* − 1). The correct midday time is thus obtained. Figure 7 depicts the effect of this modification.

This strategy allows the coordinates having the sunset of one day and the sunrise of the next day to be found on hardware platforms where the UTC days are treated independently. A small error is introduced, since the times of two different days are used. This error is practically insignificant since the difference of a sunrise (or sunset) times of two consecutive days is only a few minutes and by itself lower than the error of the light sensors used to detect sunrise or sunset times.

## 4. Experimental Results

In order to evaluate the feasibility, reliability and accuracy of the proposed system tests have been carried out. These tests comprised measuring the light intensity throughout the entire day and later analyzing the data, obtaining the sunrise and sunset times and then applying the algorithm explained in Section 2.2.

The sunlight measures were obtained from the NOAA Public FTP server [26]. This dataset comprise data dating back to 1995. Each entry documents the date, UTC time and sun variables values such as downwelling and upwelling global solar radiation, direct radiation, diffuse radiation, infrared, atmospheric pressure, *etc*. The files also provide the coordinates and elevation of the station above sea level where the measures were obtained. These are not underwater measurements, but serve as a realistic substitute.

Only the downwelling global solar radiation was used in the experiment, since it is the most representative of light intensity combining both direct and diffuse radiation. Furthermore, radiation is the one which the most low cost sensors measure. The downwelling global solar radiation is expressed in *W/m*^2^ where theoretically the minimum value is 0. However, the sensors employed by NOAA are thermopile-based, and it is not unusual for these solar instruments to register small negative signals at night. This offset is attributed to the thermopile cooling to space.

### 4.1. Identifying Sunrise and Sunset Times

The problem of accurately identifying the sunrise and sunset times is difficult using only light intensity data. Comparing the measured data with the times given by the NOAA celestial model, it can be assumed that when the data is close to 0 *W/m*^2^ a transition occurs, that is, a transition from day to night, or vice versa. Using such a threshold to identify the sunrise and sunset times is a very simple solution, and its accuracy depends on the atmospheric conditions for that day since a cloudy day the light intensity value will be lower at the same hour than for a sunny day. However, due to the measurement system offset, setting a threshold of 0 gives accurate sunrise and sunset measurements. Of course, this threshold depends on the hardware employed and should be calibrated.

**Figure 8.**
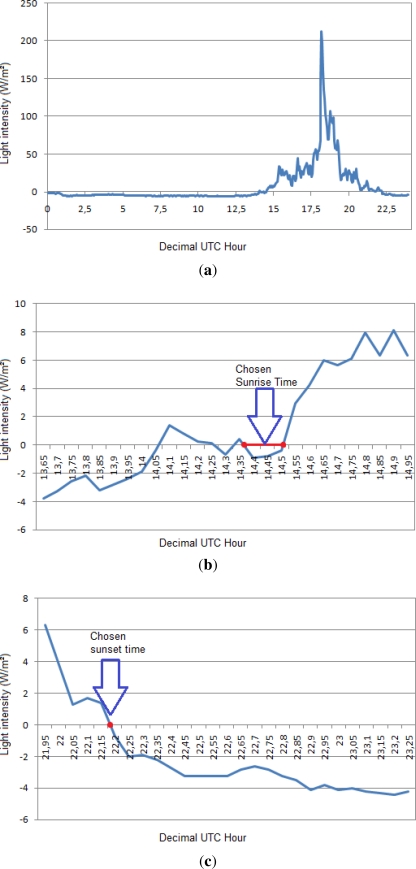
Sunrise and sunset times identification (threshold = 0). (**a**) Real NOAA light intensity data for one day; (**b**) Sunrise transitions and how the sunrise time is chosen; (**c**) Sunset transition. In this case there is only one transition.

However, due to signal noise, using a simple zero crossing condition is not enough, as it may result in many transitions per day. For days with more than two transitions, the mean time of the two transitions closest to those of neighbouring days are used (see Figure 8).

The strategy for handling fractional days relies on information about the previous day. When this information is not available the sunrise time for the current day is used instead.

The main steps of the approach can be summarised as follows:

- The celestial model used is described in Section 2.2.
- The downwelling global solar radiation measurements available at the NOAA FTP server are used.
- Each day is treated independently of each other.
- A time window based algorithm is employed to discover the sunrise and sunset transitions.
- The equinoxes have not been taken into account when running the program (days 81, 82, 265 and 266 for each year).

### 4.2. Results Analysis

**Figure 9.**
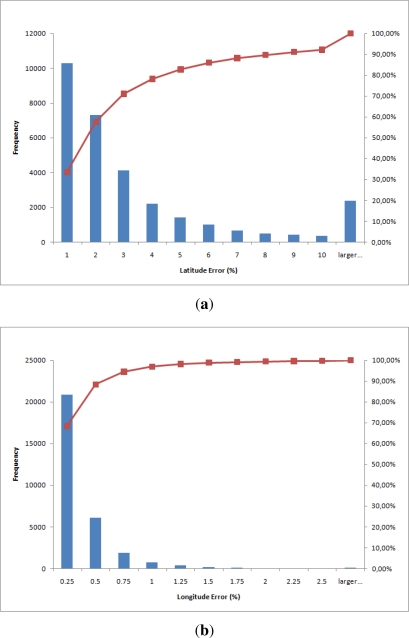
Histograms showing the absolute percentage error. (**a**) Latitude histogram; (**b**) Longitude histogram.

Figure 9 shows the error histograms for latitude and longitude using the equation of time correction. They show the absolute error in percentage in the X axis and in the Y axis the number of measures in which the error is given by the X axis. The error percentage for the latitude is:

$$e_{\mathrm{latitude}}\%=\frac{e_{\mathrm{latitude}}}{180}\;100$$

Similarly, for the longitude:

$$e_{\mathrm{longitude}}\%=\frac{e_{\mathrm{longitude}}}{360}\;100$$

The interpretation of these histograms is that most days only have a small error. For instance, more than 10.000 days (almost 35% of the total number of tested days) had less than 1% error in longitude. The red line shows the accumulated percentage. The errors for longitude is less than those for latitude.

**Figure 10.**
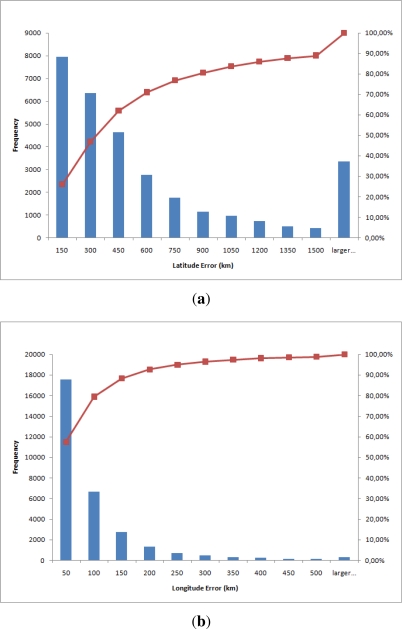
Histograms showing the absolute error in kilometers. (**a**) Latitude histogram; (**b**) Longitude histogram.

**Figure 11.**
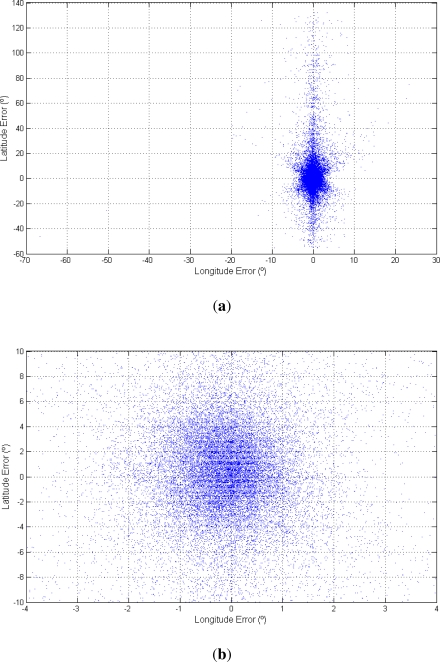
Dispersion plots of the results. (**a**) Total dispersion plot; (**b**) Dispersion plot focused on the origin.

Figure 10 shows the same histograms as in Figure 9 but with kilometers as unit converted using 1°= 111.12 kilometers in latitude and 1°= 111.12 cos *φ* kilometers in longitude. Moreover, Figure 11 shows a dispersion plot with the error obtained for each day. The results obtained prove that the algorithm works well using only sunlight intensity data. However the error in kilometers is high for most common applications. The dispersion plot (Figure 11) shows that most points are close to the origin. The longitude accuracy is high while the latitude accuracy is somewhat worse, though acceptable. Thus, the dispersion plot is concentrated in a vertical line due to the equinoxes.

**Figure 12.**
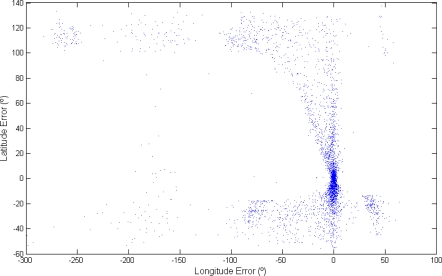
Dispersion plots of the results for days with noisy measurements.

Next, Figure 12 shows results from days where the sunlight signal is noisy and where there were more than two transitions per day. Here, the points are concentrated around zero error and in a vertical line. However, in this case the accuracy is not as high as in the previous examples.

**Figure 13.**
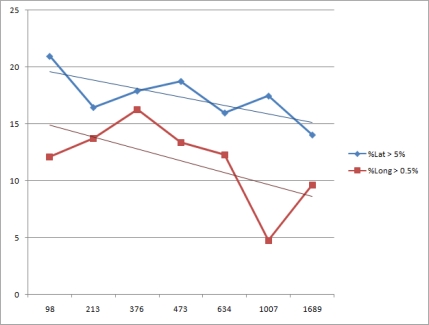
Error percentage against elevation of the stations.

Figure 13 also reveals how the error percentage for a given value (manually chosen) varies among different stations as a function of the elevation over the sea level. This image also includes a linear regression plot. Although there too few stations to reliably assess the tendency, it is possible to assert that the higher the station the lower the error. If a station is higher, it will *see* the sunrise earlier (or the sunset later). This can be translated in terms of decreasing the threshold values (Section 4.1) in function of the height of the sensor over the sea level. This has the same effect as the height: anticipating the sunrise or delaying the sunset. But this parameters depends on the light sensor employed and it also requires a height sensor.

**Figure 14.**
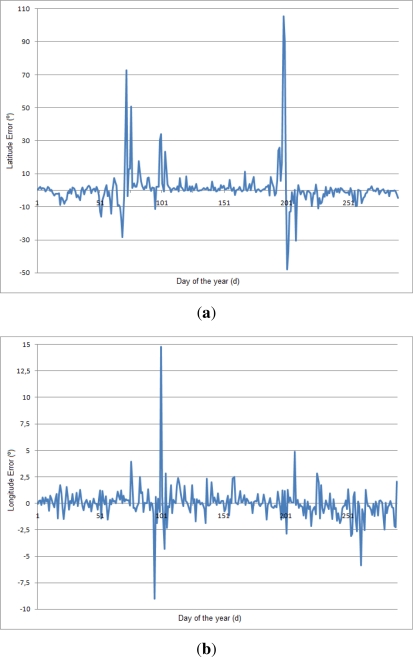
Error of the algorithm for all days of 2010. (**a**) Latitude error; (**b**) Longitude error.

Finally, Figure 14 shows the error of the algorithm for 2010 data for one of the stations. It is interesting to compare this graph with Figure 6. It is possible to see how the equinoxes increases the error enormously in the latitude. On the other hand, the longitude error points out that the error of the system is higher than the error introduced because of the Spencer EqT. Thus, the critical part of the algorithm is to identify accurately the sunrise and sunset times.

## 5. Discussion

Most global localization methods are based on GPS [27]. However, the GPS technology is problematic.

On the other hand, the SGPS is a stand-alone system that is not dependent on any technical infrastructure. It requires a modest hardware and simple software. SGPS is a low cost system with a prototype cost of less than 40 dollars (30 euros).

SGPS system is energy efficient. The energy consumption depends mainly on the number of measures taken. The system only need to take measurements during sunrises and sunsets, and the system can remain asleep the rest of the time.

On the down side, the accuracy of the SGPS accuracy is low compared with other systems. Moreover, the position refresh rate is lower than for other systems. For instance, GPS or GSM can refresh the position every few seconds.

Another drawback is that the object must be stationary and the system does not work properly during the equinoxes, which means that there are several days in which the results are not reliable at all. However, this problem can be solved by interpolation as it is well known when equinoxes occur.

The system will not work beyond the Northern or Southern polar circles during the summer or winter seasons since there are no sunrises or sunsets. More specifically, the limitation implies that the system can be used only when there is a sunrise or sunset in those latitudes which satisfy the following conditions:

−90°+δ<φ<90°−δ

during the Northern Hemisphere summer, or:

−90°−δ<φ<90°+δ

during the Northern Hemisphere winter. In both equations *δ* is the sun declination and *φ* the latitude. Out of the latitudinal ranges it is total daylight or total nighttime.

## 6. Conclusions and Future Work

This paper presents a novel global localization system, based on measuring the cyclic variations in light intensity resulting from the movement of the celestial bodies. In this case, the rotation of the earth and the resulting sunrises and sunsets are taken into account.

Thus, an algorithm is proposed to find the geographic coordinates for an outdoor object which measures the light intensity throughout a day and is able to identify accurately the sunrise and sunset times.

The conclusion obtained from the results are that the accuracy of the system is relatively high taking into account the devices which are required to build the system. Also, it is denoted that the total accuracy mainly depends on the precision when identifying sunrises and sunsets, and not on the celestial model employed.

Compared to current global localization methods, SGPS can not be applied to tasks requiring a high accuracy, such as search and rescue, navigation, *etc*. However, the accuracy should be sufficient for certain data gathering applications such as water monitoring and climate change monitoring.

Future research includes improving the accuracy using other magnitudes such light spectrum and improving robustness towards impurities in the water. Moreover, artificial intelligence techniques may be employed to better identify the sunrise and sunset times. Another improvement is to study ways of counteracting the systematic error in longitude due to the usage of the Spencer EqT and latitude due to the equinoxes.
